# Protocol for randomized control trial of a digital-assisted parenting intervention for promoting Malaysian children’s mental health

**DOI:** 10.3389/fpsyg.2022.928895

**Published:** 2022-09-23

**Authors:** Nor Sheereen Zulkefly, Anis Raihan Dzeidee Schaff, Nur Arfah Zaini, Firdaus Mukhtar, Noris Mohd Norowi, Rahima Dahlan, Salmiah Md. Said

**Affiliations:** ^1^Department of Psychiatry, Faculty of Medicine and Health Sciences, Universiti Putra Malaysia, Serdang, Malaysia; ^2^Department of Multimedia, Faculty of Computer Science and Information Technology, Universiti Putra Malaysia, Serdang, Malaysia; ^3^Department of Community Health, Faculty of Medicine and Health Sciences, Universiti Putra Malaysia, Serdang, Malaysia

**Keywords:** online parenting intervention, parenting, children mental health, digital-assisted intervention, Malaysia

## Abstract

**Background:**

Mental illness among Malaysian children is gradually reaching a fundamentally alarming point as it persistently shows increasing trend. The existing literature on the etiologies of children’s mental illness, highlights the most common cause to be ineffective or impaired parenting. Thus, efforts to combat mental illness in children should focus on improving the quality of parenting. Documented interventional studies focusing on this issue, particularly in Malaysia, are scarce and commonly report poor treatment outcomes stemming from inconvenient face-to-face instructions. Consequently, proposing an accessible online and digital-assisted parenting program is expected to reach a larger number of parents, as it can overcome substantial barriers. Hence, this study aims to develop a universal digital-assisted preventive parenting intervention called DaPI, that aims to enhance mental health of children in Malaysia.

**Methods:**

A total of 200 parents of children aged 10–14 years will be recruited and randomized into two groups either intervention or waitlist-control based on a 1:1 ratio for a duration of 8 weeks. Those in the intervention group will receive eight sessions of the DaPI program that focus mainly on parenting and children’s mental health. The primary outcome of this study will essentially focus on the changes in parent-reported parenting behavior and parental self-efficacy. The secondary outcome will be changes in children’s mental health (i.e., behavioral problems and emotional maladjustment). Assessments will be arranged pre- and post-intervention as well as at the 1-month follow-up. Analyses will be conducted using a paired *t*-test and multivariate analysis of covariance.

**Discussion:**

The expected outcome will be the establishment of DaPI in promoting children’s mental health by targeting changes in parenting behavior and parental self-efficacy in Malaysia. Findings from this study will be beneficial for policymakers to invest in parenting programs that could provide support to parents in enhancing their child’s overall development.

**Clinical trial registration:**

[www.irct.ir], identifier [IRCT20211129053207N1].

## Introduction

### Background

The dramatic rise of mental health problems among the younger generation in Malaysia is drawing great concerns from various societal groups, particularly parents, educators, researchers, and community leaders for its potential detrimental impact on the nation’s socio-economic development (IPH, 2017). Depression, anxiety, stress, and disruptive behaviors are typical mental health problems that are seen in children. These problems can develop as early as 10 years-old and can be evident as children go through the stages of childhood development. It is thus, important for parents to be aware of the symptoms of mental health problems in children and to seek immediate treatment. Untreated childhood mental health problems can evolve into major mental disorders that can persists into adulthood thereby affecting an individual’s adult life.

To combat this rising issue of mental health problems in Malaysian children, preventive and interventional strategies need to be implemented. Parenting programs would have the potential to both prevent and reduce the risk of mental health problems in children. Developing effective parenting programs requires efficient frameworks to be in place (Bowlby, 1969; Bandura, 1977; Belsky, 1984; Baumrind, 1991) that target changes in parenting practices to promote desirable child outcomes (Sanders and Kirby, 2015; Baker, 2016). Intervention programs for parents can vary from universal to selective or even indicated prevention (Leslie et al., 2016; Lindsay and Totsika, 2017). Universal intervention is offered to all parents seeking improvement in parenting practices or those in need of support, regardless of the risk level. Selective intervention targets parents of children who are at high risk and have not been referred to a clinic. The indicated intervention serves parents of children who have been referred to clinics to seek professional help for their mental health problems.

Despite evidence of the effectiveness of parenting programs, engaging parents and retaining them in the programs is a challenge. Parent attendance rates have been shown to vary depending on numerous factors, from scheduling (Shenderovich et al., 2018) to parental stress (Chacko et al., 2017) and their attitude (Ozbek et al., 2019). As these interventions are traditionally conducted face-to-face, parents may find it challenging to commit and engage in weekly sessions because of difficulty in balancing personal, family, and work life. Advancements in technology have enabled intervention research in the Western society to utilize digital-assisted (e.g., computers, smartphones, tablets, and the Internet) interventions to increase parents’ adherence to parenting programs. Furthermore, digital-assisted intervention helps in expanding the reach of a given treatment by allowing parents to access parenting programs at their own convenience regardless of their location and reduces resources required in implementing such programs (Hansen et al., 2019).

Parenting intervention research to encourage children’s development is rare in Malaysia, and is further scarce on mental health. A few prior studies (e.g., Mohamed and Hussin, 2015; Ahmad et al., 2018) have designed research programs mainly *via* face-to-face interventions either at home or at a clinic. Nonetheless, a study used social media to support face-to-face interventions in their parenting program to reduce obesity in children (Ahmad et al., 2018). Ahmad et al. (2018) in their study revealed that adherence was higher among parents participating in social media than among those participating in traditional face-to-face sessions. This implies that Malaysian parents are becoming more adherent to digitally assisted interventions compared to the traditional approach.

### Mental health in children

Mental health problems in children, especially in those aged 10–14 years, are becoming more prevalent (IPH, 2020). Generally, the common mental health problems reported in children can be categorized into behavioral (e.g., antisocial and conduct behavior), developmental (e.g., autism and global developmental delay), and emotional disorders (e.g., depression and anxiety) (Gould, 2016). The Malaysian National Health Morbidity Survey reported that the general prevalence of mental health problems among children aged 5–15 years was 12.1% (IPH, 2015). The survey further revealed that the prevalence of mental health problems caused by peer-related problems (32.5%) was the highest, in contrast to conduct problems (16.7%), emotional problems (15.7%), prosocial skills (11.2%), and hyperactivity (4.6%) (IPH, 2015). In 2017, a survey (IPH, 2017) showed that anxiety (39.7%) had the highest prevalence, unlike depression (18.3%) and stress (9.6%) for children aged 13–17 years. Anxiety is thus a more predominant mental health problem among Malaysian school-going children than depression and stress.

Children suffering from mental health problems pose longstanding consequences for families, society, and the country at large. In most cases, children’s mental health problems go undetected or unacknowledged and are left without any intervention. This increases the risk of growing up and developing an array of difficulties involving and not limited to personal and family relations; academic achievement; and emotional, physical, and behavioral health (Sheehan, 2017). If mental health problems in children are unsuccessfully managed, they persist throughout their adult lives. For example, persistent and recurrent depression and anxiety in children may manifest as a major depressive disorder or generalized anxiety disorder (DSM-5; American Psychiatric Association, 2014). It is clear that early childhood intervention in mental health is essential for ensuring children’s optimal development. Given the increase in prevalence and societal concerns regarding children’s mental health problems, research has begun to focus on developing effective early prevention programs to curb mental health problems in children. As parents are salient figures throughout the developmental stage of their children, improving parenting practices will help enhance their mental health (Melchior and van der Waerden, 2016).

### Digital parenting intervention for children’s mental health

Emerging research has suggested the use of digital-assisted interventions to address various barriers in parenting interventions (Flujas-Contreras et al., 2019; Hansen et al., 2019; Harris et al., 2020; Hamdani et al., 2021; Peyton et al., 2022). Digital-assisted interventions show great potential in fostering the mental health of a community due to better reach and efficiency of mental healthcare delivery. Smartphone apps, short message services (SMS), social media, and interactive websites are widely utilized to support behaviors involved in prevention, self-management, and delivery of evidence-based mental health care practice (Stawarz et al., 2019; Marshall et al., 2020; Hamdani et al., 2021). In comparison to conventional face-to-face intervention, digital-assisted intervention allows treatment and services to be more accessible to parents, anytime and anywhere, and are available 24/7 upon demand to suit parents’ schedules (Baumel et al., 2016).

Digital-assisted parenting interventions delivered online may be self-directed (where parents complete a program without any assistance from a practitioner) or may provide guidance and support from a practitioner *via* various methods (i.e., phone calls, video conferencing sessions, or text messages and emails). Whether self-directed or assisted, both provide clear benefits for parents. Nevertheless, parents in self-directed online intervention may not be able to practice parenting skills learned in front of a practitioner, nor have their progress throughout the program, reviewed. In view of such setbacks, recent online interventions have begun to include some form of practitioner support and are not fully self-directed in nature (Tully et al., 2017). Despite the effectiveness and acceptability of various types of digitally assisted interventions established in Western culture, such interventions specifically related to parenting and children with mental health issues in the Malaysian context are yet to be established. Moreover, the few available interventions have focused on limited developmental aspects (e.g., reducing obesity) (Ahmad et al., 2018) rather than mental health. Furthermore, in this context, only a study by Ahmad et al. (2018) utilized social media alongside traditional face-to-face interventions. They reported that among 122 parents, the majority participated better through social media (96.9% WhatsApp and 81.3% Facebook) compared to face-to-face sessions (42.2 and 68.8%, respectively). This suggests that Malaysian parents with young children are more likely to favor digitally assisted interventions that are more easily accessible to them than traditional methods of interventions.

In recent years, the pattern of digital usage among Malaysians has increased steadily. In its latest report, the Malaysian Communication and Multimedia Commission (MCMC; 2020) indicated that a vast majority (88.7%) of Malaysians utilized the Internet daily, spanning over a period of 1–4 h (32.9%). The highest number of Internet users were males (54.3%) and those in their 30 s (25.9%) and 40 s (17.9%), who, on average, spent a longer time (i.e., between 5.9 and 7.3 h) on the Internet daily. A majority (88.6%) of Malaysians used the Internet at home, while 68.1% tended to use the Internet while on-the-go, and slightly more than half (56.4%) of the users accessed the Internet at the office. Malaysian users were found to use smartphones (93.1%) when accessing the Internet, followed by laptops (44.2%), desktops (28.1%), and tablets (20.4%). The online activities frequently performed by Malaysians were mainly communication (96.5%), visiting social networking platforms (85.6%), and acquiring information (85.5%). Given the encouraging figures of Internet usage by Malaysians, an online digital-assisted parenting program would most likely be a more promising platform for Malaysian parents to receive parenting education and skills in encouraging a positive mental health of their children.

As parents are active agents in influencing child development, their participation in parenting intervention programs would encourage improvement in parenting and consequentially reduce behavioral and emotional problems in children. Considering the significant contribution of parents in child development and emerging preference for digital-assisted intervention, this paper presents a protocol for a universal, digital-assisted preventive parenting intervention called “DaPI,” with the aim of supporting Malaysian parents in promoting their children’s mental health.

### Objective

The overarching aim of this study is to determine whether a universal, digital-assisted preventive parenting intervention named DaPI will be effective in promoting the mental health of children by targeting parenting behavior and parental self-efficacy. We hypothesized that parents in the DaPI program would report lower rates of ineffective parenting behavior and higher parenting self-efficacy (primary outcomes), which would lead to improved mental health in their child (secondary outcomes, i.e., better behavior and emotional adjustment).

## Methods and analysis

### Trial design

This study is a randomized controlled trial (RCT) of the DaPI online program. Interested Malaysian parents with children aged 10–14 years will be randomly assigned to either the intervention group (DaPI) or waitlist control (WLC) group. This study will experimentally evaluate the relative impact of DaPI and WLC on parenting and child outcomes. The SPIRIT (Standard Protocol Items: Recommendations for Interventional Trials) guidelines for RCT were used in this study.

Self-reported data will be collected at three time points (pre- and post-intervention and 1-month follow-up). Parents will need to sign an online consent form before completing the pre-intervention (baseline) questionnaire on enrollment in the study. Parents will be able to access the online program at their own pace throughout the span of 8 weeks and then complete a post-intervention questionnaire. Parents will complete the follow-up questionnaire a month after answering the post-intervention questionnaire.

### Eligibility criteria

Eligible participants will be parents of children aged 10–14 years who are currently living in Malaysia. We aim to recruit 200 parents to participate in the study. Parents from the same household can participate if they focus on a different child within the stipulated age range. It would be an added value to the household if both parents took the opportunity to upgrade their parenting knowledge and skills to improve their children’s development. The inclusion criteria for parents receiving the DaPI would be to have a child within the specified age range and can understand the Malay language. In terms of exclusion criteria, parents who specified that they are currently receiving professional help for their child’s mental health problems will be excluded. No other screening assessments for inclusion or exclusion of the DaPI program will be conducted.

### Participants

#### Randomization

Participation randomization to either the DaPI or WLC group will be overseen by a research coordinator. Using a simple random technique, each participant will be assigned a unique identification number generated by a random number generator in a 1:1 proportion. Immediately after the participants completed the pre-intervention questionnaire, the research coordinator will notify them of their allocation *via* email.

**FIGURE 1:**
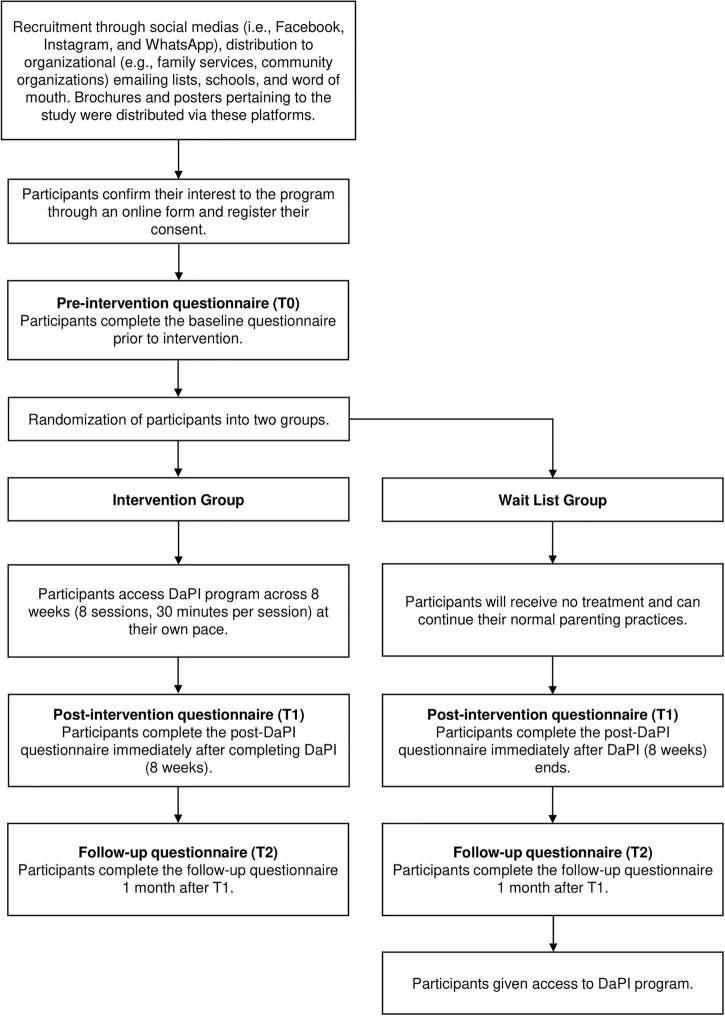
Participant flow through the study.

Participants in the DaPI group will get immediate access to the online program, by logging in with their user ID and password provided by the researcher. They will be able to access the program as per their convenience particularly, their chosen time and day. Meanwhile, participants in the WLC group will be given access to the program after the 1-month follow-up questionnaire has been collected. Figure 1 summarizes the flow of participants in this study. Due to the nature of the intervention, participants and researchers directly involved in the online intervention cannot be blinded to the allocation. Nonetheless, researchers involved in data collection and the analyses will be blinded, as they are not involved in participant allocation or online intervention.

#### Study retention

One of the challenges in an intervention study is to retain participants, particularly those allocated to the WLC group. Parents allocated to the WLC group may find it difficult to wait or withdraw from the study by the time they are offered the DaPI program. Parents in both the DaPI and WLC groups will be required to complete questionnaires at three different times (pre- and post-intervention, and 1 month follow-up). Parents in the DaPI group will be required to complete the online program within 8 weeks, whereas those in the WLC group will have a 12-week delayed access to the program. As a means to increase retention, the present study will give parents in both groups an e-voucher worth MYR10 as an incentive for every time they completed a questionnaire (Dumas et al., 2010; Brueton et al., 2017). Parents who withdrew from the study will be contacted to determine the reason for their withdrawal from the program.

#### Sample size and power analysis

A power analysis using the G*Power software (Faul et al., 2007) will be performed in estimating the required sample size. To detect a medium effect size of 0.50, with alpha set at 0.05, and power of 0.80, a minimum sample size of 128 is required. Based on recommendations from Hall and Bierman (2015) review of high attrition from technology-assisted parenting intervention, this study will allow an attrition of 50%, making the target sample size 200, with 100 participants for each group.

#### Recruitment

Participants will be recruited across Malaysia through social media (Facebook, Instagram, WhatsApp), government and non-government organizations’ emailing lists, schools, word of mouth, and the study website. Brochures and posters pertaining to the study will be distributed *via* social media and their respective organizations. Consent will be obtained from parents and their respective bodies in the organization prior to their distribution to its members. Interested parents will be able to contact the research team based on contact information on the flyers. Once parents meet the study criteria, they will be provided a link to the online questionnaire. Parents who do not wish to participate or withdraw from the intervention may do so by exiting the online survey and not responding to weekly session reminders.

#### Intervention

DaPI is an adaptation of established evidence-based Western parenting programs, such as Parenting Wisely (Gordon, 2003), Triple-P Positive Parenting Program (Sanders et al., 2000), and the Incredible Years (The Incredible Years, 1984), that focused on parental factors associated with children’s mental health in Malaysia. Considering the diverse ethnicity and religious culture of Malaysia, a parenting module incorporating culturally specific parenting beliefs with local examples would be more suitable for Malaysian parents. As emphasized in previous literature (Hong et al., 2012; Keshavarz et al., 2013; Andrew, 2019; Zulkefly et al., 2021), the parenting beliefs of Asian parents differ from those of the Western construct. For example, Western parents view authoritarian parenting as negative, harsh, and more likely to yield negative outcomes for children. Contrastingly, Asian parents view an authoritarian style as positive style of parenting, where they show care, concern, and involvement through firm control and governance of the child. Furthermore, Asian parents also expect their children’s filial piety and obedience to their families. Contrary to their counterparts from the West, children of authoritarian parents show positive outcomes and feel warmth and care through their parents’ control and firm parenting behavior. Therefore, adjustments and adaptations are needed when using Western programs in the Asian context.

The DaPI can be completed online using a computer, tablet, or smartphone at any time, which is convenient for the parents. The interactive online program will consist of various materials including written information, pre-recorded videos, audio, interactive quizzes, and family activities across the eight sessions. Table 1 presents an overview of each session in the DaPI. The DaPI program will be conducted in Malay, the official language of Malaysia, and will use examples and pictures that represent Malaysian local culture.

**TABLE 1:** Sessions content.

Session number and title	Content overview
1. Normal development and challenges in early adolescence	• Developmental characteristics of early adolescents. • Developmental challenges of early adolescents.
2. Parent-child attachment	• Role of parent-child attachment in child mental health development. • Principles of building secure parent-child attachment relationship.
3. Effective communication	• Role of effective communication in promoting mental health of child. • Principles of effective communication.
4. Parenting behavior and challenges	• Types of parenting behavior. • The effects of parenting behavior on child mental health. • Overcoming parenting challenges.
5. Family cohesiveness	• Role of family cohesiveness. • Strategies to create family cohesiveness.
6. Family conflict	• Overcoming parent-child conflict.
7. Parent mental health	• Negative thinking in parenting • Emotional challenges in parenting • Strategies to challenge parental stress.
8. Child mental health	• Signs and symptoms of child mental health problems. • Helping child overcome negative thinking. • Strategies to help children manage stress.

Parents in the DaPI group will be instructed on effective parenting skills using demonstration, quizzing, recognition, and feedback on correct or incorrect answers *via* an interactive online program. For example, parents will be given a scenario of an adolescent problem and will need to choose one of several solutions, only one of which is an effective method for dealing with this problem. Several review questions will be asked to reinforce the skills learned further.

Session 1 of DaPI will be immediately accessible to parents once they are provided with their login details. New sessions will be available to parents each week in a sequential order upon their completion of the previous session. For example, if a parent is in Week 3 of the DaPI program, the program will unlock session 3 for the parent. However, parents can only start session 3 once they have completed session 2. They are also encouraged to complete one session every week throughout the 8-week program. Each session will take approximately 30 min to complete. Parents will also have the option of saving their progress and returning to continue their sessions at any time, convenient to them. Additionally, parents could self-monitor their progress and receive written feedback from an experienced clinical psychologist on their activities throughout the eight sessions. They can also request support in implementing the intervention techniques from the clinical psychologist by clicking the “chat with me” button.

Several strategies will be employed to engage parents in the DaPI program throughout the 8-week intervention period. Based on earlier studies of online and digital-assisted interventions, a program with frequent reminders and new weekly content would have greater participant usage (Brouwer et al., 2008; Kelders et al., 2012). Automated text messages *via* WhatsApp, SMS, and email will be sent to the participants after completing each module to reinforce the activities. The automated messages also will notify parents of the availability of a new session and prompt them to use the program. Sessions will be considered complete when parents view the summary page of a particular session. Booster reminder emails will be sent after 4 weeks in the program and 1 week before the program ends. If parents in the DaPI program become inactive for a period of 3 weeks, a text message will be sent to check if they are experiencing any technical issues and to boost their interest in continuing with the program.

#### Control

Participants in the WLC group will receive no treatment and will continue with their usual parenting practices in managing their children. After a 1-month follow-up, the participants will be given access to the DaPI program.

#### Outcome measures

Parents in both the DaPI and WLC groups will complete a questionnaire comprising the following questions at pre- (baseline) and post-intervention (8 weeks) and at the 1-month follow-up mark. Parents in the DaPI group will be allowed to complete the questionnaire, regardless of whether they have completed all eight sessions of the DaPI program. Prior to answering the questionnaire, parents with more than one child within the required age range will need to select a child as the focus of the study. If both parents in the same household wished to participate, they would choose a different focus child within the stipulated age range. The questionnaire will be administered in Malay. A forward backward translation procedure will be conducted for questions that have not been translated and validated in a Malaysian sample.

#### Primary outcomes

##### Parenting measures

(a). Parenting behavior—Parenting behavior of parents will be measured using a self-report multidimensional assessment of parenting scale (MAPS; Parent and Forehand, 2017). This 34-item scale comprises two broad domains (i.e., Positive and Negative Parenting) and seven narrowband domains of parenting practices. Out of the seven narrowband domains, four (i.e., Proactive Parenting, Positive Reinforcements, Warmth and Supportiveness) are in the positive parenting domain, while three (i.e., hostility, physical control, and lax control) in the negative parenting domain. Items are rated on a 5-point Likert scale ranging from 1 (never) to 5 (always). Total scores are obtained by summing up all items in the respective domains, where higher scores on the positive parenting domain indicate higher levels of warmth, supportiveness, and good positive control. Contrarily, higher scores on the negative parenting domain reflect higher levels of hostility as well as both over- and under-control. The internal consistency for both the positive and negative parenting dimensions are reported to be strong, with α = 0.90 and 0.88, respectively (Parent and Forehand, 2017). Similarly, all seven (7) narrowband domains reported good to excellent reliability ranging from 0.77 to 0.91 (Parent and Forehand, 2017).

(b). Parental self-efficacy–Parental self-efficacy will be measured using the 20-item subscale of the Child Adjustment and Parent Efficacy Scale (CAPES; Morawska et al., 2014). Parents will rate their self-efficacy in managing their child’s behavioral and emotional health over the last 4 weeks using a 4-point scale ranging from 0 (not true of my child at all) to 3 (true of my child very much, or most of the time). The total scores will be obtained by the summation of all items, where higher scores indicate better self-efficacy in dealing with children’s mental health. This subscale has been reported to have good internal consistency, with a Cronbach’s alpha of 0.96 (Baker, 2016).

#### Secondary outcome

##### Children mental health measure

Parents’ perceptions of their children’s mental health will be based on the 30-item Child Adjustment and Parent Efficacy Scale (CAPES; Morawska et al., 2014). This scale comprises two subscales that assess children’s behavioral problems and emotional maladjustment. Parents will rate their child’s behavioral problems and emotional maladjustment over the last 4 weeks on a scale ranging from 0 to 3 (0 = not true of my child at all; 3 = true of my child very much, or most of the time). The total score will be calculated by summing all 30-items (score range 0–90), where higher scores indicate a higher intensity of problems. The CAPES has been found to have good internal consistency (α = 0.90). Similarly, the behavioral subscale (α = 0.90) and emotional maladjustment (α = 0.74) were found to have a high level of reliability (Morawska et al., 2014).

#### Other measures

##### Personal characteristics

Information on parents’ personal characteristics, such as age, education, occupation, marital status, race, self-esteem, and mental health (i.e., depression, anxiety, and stress) will be monitored.

(a) Self-esteem: Parents’ self-esteem will be measured using the Rosenberg Self-esteem Scale (Rosenberg, 1965). This scale comprises 10 items and rated on a 4-point Likert scale ranging from “strongly disagree” to “strongly agree.” Total scores will be obtained by summing all items after reverse-scoring negatively worded items. Higher scores indicate higher self-esteem. The internal consistency of this scale has been reported to be excellent (α = 0.92; Rosenberg, 1965).

(b) Mental health: Parents’ symptoms of depression, anxiety, and stress will be assessed using the Malay version of the Depression Anxiety Stress Scales-21 (DASS-21; Musa et al., 2007). Parents will rate the extent to which items were applied to them over the past week on a 4-point scale ranging from “did not apply to me at all’ to “applied to me very much, or most of the time.” Higher scores will suggest poorer mental health. The internal consistency for the overall scale was reported to be very good, with a Cronbach’s alpha of 0.90. Similarly, all subscales had good internal consistency (α ^Depression^ = 0.84, α ^Anxiety^ = 0.74, α ^Stress^ = 0.79) (Musa et al., 2007).

##### Child characteristics

Information on the focus child’s personal characteristics, such as age, sex, and education will be focused on.

##### Family social context

Information on family characteristics such as family income, number of children, family structure (intact/non-intact), and family relations (i.e., parent-child conflict and family cohesiveness) will also be dealt with.

(a) Parent-child conflict—Parents’ perception of the intensity of their parent–child conflict will be assessed using the 6-item short version of the Network of Relationships Inventory (Furman and Buhrmester, 1985; De Goede et al., 2009). Parents will indicate their answers on a five-point Likert scale (ranging from 1 = a little or not at all to 5 = more is not possible), with higher scores indicating higher levels of intensity of the parent-child conflict. Cronbach’s alpha for this scale has been reported to be high ranging from 0.90 to 0.92 (Furman and Buhrmester, 1985; De Goede et al., 2009).

(b) Family cohesion: Parents will rate family cohesion using a 9-item scale developed by Tolan et al. (1997). This scale measures how well family members communicate with each other, are emotionally close, dependent, and are supportive of one another. The parents will rate the items using a 4-point Likert scale ranging from “not true at all” to “very true.” The summation of the items indicates a higher family cohesion. Cronbach’s alpha (0.84) for this scale indicated strong reliability (Leidy et al., 2010).

##### Program use and client satisfaction measure

(a) Parent patterns of use of the DaPI program will be monitored throughout the 8-week program. At the post-intervention assessment, parents in the DaPI group will be asked to rate details about the program and comment on its content as well as features. Items are rated on a 5-point scale with responses ranging from 1 = very poor/not at all helpful/strongly disagree to 5 = very good/very helpful/strongly agree. High scores will indicate the effectiveness of the DaPI program in delivering content.

(b) Parents’ satisfaction with the DaPI program will be assessed using the eight-item Client Satisfaction Questionnaire (CSQ; Sanders et al., 2012). This scale will assess how well the program met parents’ needs, decreased dysfunctional parenting, and mental health issues of the child. Higher scores indicate higher satisfaction with the quality of service. This scale has been reported to have excellent internal consistency with a Cronbach’s alpha of 0.93 (Baker, 2016).

### Statistical analyses

Multivariate analysis of covariance (MANCOVA) will be employed to compare the mean of the outcome variables at pre-assessment, post-assessment, and 1-month follow-up between the two groups (i.e., DaPI vs. WLC). Pairwise comparisons will be performed to identify specific differences in the outcome variables between the groups. Similarly, intention-to-treat analysis (ITT) will be conducted on the data to allow all participants to be included in the analysis, regardless of whether they withdrew from the study or did not complete the intervention. Missing data will be treated using multiple imputations, which is a relatively flexible, general approach for dealing with missing data. The intervention will be considered effective if the parents in the DaPI group show more improvement over time on one of the outcome variables compared to the control group, at a significance level of 0.05, and at small to medium effect size *d* [0.2–0.5].

## Discussion

This protocol paper outlines the background and design of an online digital-assisted parenting intervention (DaPI) that aims to promote the mental health of children in Malaysia by targeting parenting behaviors and parental self-efficacy. The DaPI program was designed to be practical and interactive, yet strongly based on research evidence. This RCT will examine whether, in comparison to the control group, parents who received the DaPI program would show significant changes in parenting behavior and parental self-efficacy. Additionally, the RCT will assess whether, in contrast to parents in the WLC group, parents in the DaPI program would report significant changes in their children’s behavioral problems and emotional maladjustment. Overall, it is expected that participating in the DaPI program will improve parenting behavior and confidence as well as reduce children’s behavioral problems and emotional maladjustment. Better knowledge and skills in parenting will help to improve parents’ interaction with their children in an intellectually stimulating and emotionally reassuring way. Thus, making children more emotionally, cognitively, and socially able to manage challenges.

This RCT is timely considering the widespread use of digital devices across the Malaysian population. Due to the COVID-19 pandemic, digital-assisted parenting intervention would be more attractive for parents who are not keen on traditional face-to-face intervention. Moreover, a universal, online, preventive parenting intervention is deemed to be more favorable for working Malaysian parents who would like to receive additional support on parenting practices but have difficulty attending parenting interventions during their working hours. An online intervention such as DaPI would allow anonymity to parents to participate without fear of mental health stigma or judgment regarding their parenting skills. Furthermore, if found effective, the DaPI program can be widely disseminated as a cost-effective universal preventive parenting program for all Malaysian parents, leading to better mental health, quality of life, and overall development for their children.

Improved parenting behavior and parenting self-efficacy can reduce children’s use of primary care services (McGilloway et al., 2014) which would lead to potential cost savings. This could potentially help healthcare professionals focus more time and resources on clinical or at-risk populations. These benefits may prompt advocates and policymakers to explore the promise of and invest in preventive parenting programs to reduce the negative and costly outcomes of mental illness in children. If investments are made in preventive parenting programs, children would have more opportunities to grow to be healthy, protected, and well-developed, thus becoming productive human resources for the progress and development of a nation.

## Ethics statement

The studies involving human participants were reviewed and approved by Universiti Putra Malaysia’s Ethic Committee for Research Involving Human Subjects (JKEUPM-2021-161). The participants provided their written informed consent to participate in this study.

## Author contributions

NSZ was the principal investigator of the trial and was primarily responsible for the funding application, design, and development of the RCT and also wrote the manuscript. ARDS contributed to the literature search, web design, web monitoring, data collection, and the manuscript. NAZ aided in the web design and web monitoring, program implementation, and data collection. FM contributed to the study design and data collection. NMN participated in designing the DaPI web system and collecting the data. RD and MSS contributed to data collection. All authors have read and approved the final version of the manuscript.
